# Supplementary material for: Early postoperative alignment and preoperative stereopsis as determinants of success in intermittent exotropia surgery
Source: PLoS One. 2025 Oct 10;20(10):e0329609. doi: 10.1371/journal.pone.0329609 (PMC12513599; doi:10.1371/journal.pone.0329609)
Supplement: S1 — (DOCX) [file pone.0329609.s001.docx]

STROBE Checklist for Observational Cohort Study

| Section and Item | Item number | Recommendation | Manuscript Response / Location | Reported on Page No |
| --- | --- | --- | --- | --- |
| Title and abstract | 1 | Indicate the study's design with a commonly used term in the title or the abstract | Included in title and abstract. Study design (retrospective cohort) clearly mentioned. | 1-2 |
| Introduction  -Background/rationale | 2 | Explain the scientific background and rationale for the investigation being reported | Scientific rationale and background for IXT surgery outcomes are thoroughly described. | 3 |
| Objectives | 3 | State specific objectives, including any prespecified hypotheses | Clearly stated: to determine factors associated with surgical success in IXT. | 3 |
| Study design | 4 | Present key elements of study design early in the paper | Described as a retrospective cohort study in both abstract and methods. | 3-4 |
| Setting | 5 | Describe the setting, locations, and relevant dates | Tertiary care center in Thailand; data from 2013 to 2023 provided in Methods. | 3-4 |
| Participants | 6 | Give eligibility criteria and sources and methods of selection of participants | Inclusion/exclusion criteria and participant flow well defined in Methods. | 4 |
| Variables | 7 | Clearly define all outcomes, exposures, predictors, potential confounders | All main variables, including success definitions and stereopsis, are clearly defined. | 4-6 |
| Data sources/measurement | 8 | Give sources of data and details of assessment methods | Details of measurements (e.g., stereopsis, angle) provided. Control score data discussed as limitation. | 4-6 |
| Bias | 9 | Describe any efforts to address potential sources of bias | Bias from retrospective design discussed in limitations. Selection bias mentioned. | NA |
| Study size | 10 | Explain how the study size was arrived at | Study size based on total eligible records over study period. All eligible cases included (n=150). | 3-4 |
| Quantitative variables | 11 | Explain how quantitative variables were handled | All angles in PD, VA in logMAR; appropriate stats methods used. Details in Methods. | 4-6 |
| Statistical methods | 12 | Describe all statistical methods, including those used to control for confounding | Multivariable logistic regression used, with age added as confounder. Limitations in missing control score noted. | 6-8 |
| Participants | 13 | Report numbers of individuals at each stage | 150 patients included; no dropouts reported. All had at least 1-year follow-up. | 8-9 |
| Descriptive data | 14 | Give characteristics of study participants | Baseline demographics reported in Table 1. Age, sex, pre-op angle, etc. | 8-13 |
| Outcome data | 15 | Report numbers of outcome events or summary measures | Success and nonsuccess outcomes reported at 1 year. Shown in tables and KM plot. | 8-13 |
| Main results | 16 | Give unadjusted estimates and confounder-adjusted estimates | Adjusted ORs with 95% CI reported in Table 4. Age-adjusted model provided. | 8-13 |
| Other analyses | 17 | Report other analyses done, e.g., sensitivity analyses | No sensitivity analysis. Mentioned as limitation due to retrospective data. | NA |
| Key results | 18 | Summarise key results with reference to study objectives | Success predictors summarized clearly in Results and Discussion. | 13-15 |
| Limitations | 19 | Discuss limitations of the study | Retrospective design, lack of neurological data, and missing control scores mentioned. | 16 |
| Interpretation | 20 | Give a cautious overall interpretation | Findings interpreted cautiously in context of other literature. Clinical implications discussed. | 13-15 |
| Generalisability | 21 | Discuss the generalisability (external validity) | Generalizability discussed in limitations. Single-center nature acknowledged. | 13-15 |
| Funding | 22 | Give the source of funding and role of funders | No specific funding for this work | 17 |
