# Supplementary material for: Early postoperative alignment and preoperative stereopsis as determinants of success in intermittent exotropia surgery
Source: PLoS One. 2025 Oct 10;20(10):e0329609. doi: 10.1371/journal.pone.0329609 (PMC12513599; doi:10.1371/journal.pone.0329609)
Supplement: Attachment — Submitted filename: Response to reviewers.docx [file pone.0329609.s004.docx]

**Response Letter PLOS One**

PONE-D-25-34023

Early Postoperative Alignment and Preoperative Stereopsis as Determinants of Success in Intermittent Exotropia Surgery

PLOS ONE

Dear Dr. Srimanan,

Thank you for submitting your manuscript to PLOS ONE. After careful consideration, we feel that it has merit but does not fully meet PLOS ONE’s publication criteria as it currently stands. Therefore, we invite you to submit a revised version of the manuscript that addresses the points raised during the review process.

**Please look into the comments of all the reviewers.**

Please submit your revised manuscript by Sep 14 2025 11:59PM. If you will need more time than this to complete your revisions, please reply to this message or contact the journal office at [plosone@plos.org](mailto:plosone@plos.org). When you're ready to submit your revision, log on to <https://www.editorialmanager.com/pone/> and select the 'Submissions Needing Revision' folder to locate your manuscript file.

Please include the following items when submitting your revised manuscript:

- A rebuttal letter that responds to each point raised by the academic editor and reviewer(s). You should upload this letter as a separate file labeled 'Response to Reviewers'.
- A marked-up copy of your manuscript that highlights changes made to the original version. You should upload this as a separate file labeled 'Revised Manuscript with Track Changes'.
- An unmarked version of your revised paper without tracked changes. You should upload this as a separate file labeled 'Manuscript'.

If you would like to make changes to your financial disclosure, please include your updated statement in your cover letter. Guidelines for resubmitting your figure files are available below the reviewer comments at the end of this letter.

If applicable, we recommend that you deposit your laboratory protocols in [protocols.io](http://protocols.io/) to enhance the reproducibility of your results. Protocols.io assigns your protocol its own identifier (DOI) so that it can be cited independently in the future. For instructions see: [https://journals.plos.org/plosone/s/submission-guidelines#loc-laboratory-protocols](https://track.editorialmanager.com/CL0/https:%2F%2Fjournals.plos.org%2Fplosone%2Fs%2Fsubmission-guidelines%23loc-laboratory-protocols/1/010f01986132418c-5438627a-5149-4859-bc90-07b8441783a9-000000/0rGElk7qhM0531S-ks5QwGSbkQrObw98pk7buN6fN7U=222). Additionally, PLOS ONE offers an option for publishing peer-reviewed Lab Protocol articles, which describe protocols hosted on [protocols.io](http://protocols.io/). Read more information on sharing protocols at [https://plos.org/protocols?utm_medium=editorial-email&utm_source=authorletters&utm_campaign=protocols](https://track.editorialmanager.com/CL0/https:%2F%2Fplos.org%2Fprotocols%3Futm_medium=editorial-email%26utm_source=authorletters%26utm_campaign=protocols/1/010f01986132418c-5438627a-5149-4859-bc90-07b8441783a9-000000/ByTaBSNVBFTn23zcZRh-HvPht8IL1KdastXS5cRnfDQ=222).

We look forward to receiving your revised manuscript.

Kind regards,

PremNandhini Satgunam
Academic Editor
PLOS ONE

Journal Requirements:

When submitting your revision, we need you to address these additional requirements.

1. Please ensure that your manuscript meets PLOS ONE's style requirements, including those for file naming. The PLOS ONE style templates can be found at

<https://journals.plos.org/plosone/s/file?id=wjVg/PLOSOne_formatting_sample_main_body.pdf> and

[https://journals.plos.org/plosone/s/file?id=ba62/PLOSOne_formatting_sample_title_authors_affiliations.pdf](https://track.editorialmanager.com/CL0/https:%2F%2Fjournals.plos.org%2Fplosone%2Fs%2Ffile%3Fid=ba62%2FPLOSOne_formatting_sample_title_authors_affiliations.pdf/1/010f01986132418c-5438627a-5149-4859-bc90-07b8441783a9-000000/A-jevwyKyX4KTjKbk9LEznkchY4v70mSnAhQ5A7uK24=222)

2. We note that your Data Availability Statement is currently as follows: [All relevant data are within the manuscript and its Supporting Information files.]

Please confirm at this time whether or not your submission contains all raw data required to replicate the results of your study. Authors must share the “minimal data set” for their submission. PLOS defines the minimal data set to consist of the data required to replicate all study findings reported in the article, as well as related metadata and methods (<https://journals.plos.org/plosone/s/data-availability#loc-minimal-data-set-definition>).

For example, authors should submit the following data:

- The values behind the means, standard deviations and other measures reported;
- The values used to build graphs;
- The points extracted from images for analysis.

Authors do not need to submit their entire data set if only a portion of the data was used in the reported study.

If your submission does not contain these data, please either upload them as Supporting Information files or deposit them to a stable, public repository and provide us with the relevant URLs, DOIs, or accession numbers. For a list of recommended repositories, please see <https://journals.plos.org/plosone/s/recommended-repositories>.

If there are ethical or legal restrictions on sharing a de-identified data set, please explain them in detail (e.g., data contain potentially sensitive information, data are owned by a third-party organization, etc.) and who has imposed them (e.g., an ethics committee). Please also provide contact information for a data access committee, ethics committee, or other institutional body to which data requests may be sent. If data are owned by a third party, please indicate how others may request data access.

3. Please include captions for your Supporting Information files at the end of your manuscript, and update any in-text citations to match accordingly. Please see our Supporting Information guidelines for more information: [http://journals.plos.org/plosone/s/supporting-information](http://track.editorialmanager.com/CL0/http:%2F%2Fjournals.plos.org%2Fplosone%2Fs%2Fsupporting-information/1/010f01986132418c-5438627a-5149-4859-bc90-07b8441783a9-000000/A1ch31d9aykLsmXIqDe086h6hSrpmBF5HdZZPQFJv0w=222).

4. If the reviewer comments include a recommendation to cite specific previously published works, please review and evaluate these publications to determine whether they are relevant and should be cited. There is no requirement to cite these works unless the editor has indicated otherwise. 

Additional Editor Comments:

The reviewers have given a careful consideration and have identified areas that needs better reporting, clarity for terms used and explanation for statistical methods. I hope the authors can take these comments and work on their manuscript to improve the quality of this work.

Response: Thank you for reviewing and providing suggestions for our manuscript. We thoroughly reviewed and revised the manuscript based on your expert recommendations. We hope our revised manuscript meets your high standards and has the potential to be published in your esteemed journal.
[Note: HTML markup is below. Please do not edit.]

Reviewers' comments:

Reviewer's Responses to Questions

**Comments to the Author**

1. Is the manuscript technically sound, and do the data support the conclusions?

The manuscript must describe a technically sound piece of scientific research with data that supports the conclusions. Experiments must have been conducted rigorously, with appropriate controls, replication, and sample sizes. The conclusions must be drawn appropriately based on the data presented.

Reviewer #1: No

Reviewer #2: Partly

Reviewer #3: Yes

2. Has the statistical analysis been performed appropriately and rigorously?

Reviewer #1: Yes

Reviewer #2: No

Reviewer #3: Yes

3. Have the authors made all data underlying the findings in their manuscript fully available?

The [PLOS Data policy](http://track.editorialmanager.com/CL0/http:%2F%2Fwww.plosone.org%2Fstatic%2Fpolicies.action%23sharing/1/010f01986132418c-5438627a-5149-4859-bc90-07b8441783a9-000000/XllbaqL0BG7IoT0kSv-GYYa9sNjdfHGj0VRkXDhEdBI=222) requires authors to make all data underlying the findings described in their manuscript fully available without restriction, with rare exception (please refer to the Data Availability Statement in the manuscript PDF file). The data should be provided as part of the manuscript or its supporting information, or deposited to a public repository. For example, in addition to summary statistics, the data points behind means, medians and variance measures should be available. If there are restrictions on publicly sharing data—e.g. participant privacy or use of data from a third party—those must be specified.

Reviewer #1: No

Reviewer #2: Yes

Reviewer #3: Yes

4. Is the manuscript presented in an intelligible fashion and written in standard English?

PLOS ONE does not copyedit accepted manuscripts, so the language in submitted articles must be clear, correct, and unambiguous. Any typographical or grammatical errors should be corrected at revision, so please note any specific errors here.

Reviewer #1: Yes

Reviewer #2: Yes

Reviewer #3: Yes

5. Review Comments to the Author

Please use the space provided to explain your answers to the questions above. You may also include additional comments for the author, including concerns about dual publication, research ethics, or publication ethics. (Please upload your review as an attachment if it exceeds 20,000 characters)

Reviewer #1: Clarification of Table 3 Interpretation:
Table 3 reports postoperative alignment status at various time points grouped by 1-year surgical outcomes. However, the finding that patients with residual exotropia had higher success rates than those with early consecutive esotropia (CE) appears inconsistent with commonly observed postoperative trajectories in IXT, where early CE is typically a favorable prognostic factor. This discrepancy may confuse readers. The authors are advised to clarify this point in the Results or Discussion section and consider including a line graph showing longitudinal alignment trends (CE, orthotropia, exotropia) over time in both success and non-success groups.
Response: Thank you for your valuable suggestion. The description of the surgical success at one year postoperative was thoroughly detailed in the results and discussion sections of the manuscript, specifically on page 11, lines 211-220, and pages 14-15, lines 310-323. The longitudinal alignment trends graph is also included in Figure 2.

Definition of Sensory Success and Stereopsis Threshold:
The term “sensory outcomes were successful in 85.5% of cases” lacks a precise definition of "success." It appears the presence of any measurable stereopsis was regarded as success, but this is neither explicitly defined nor supported by comparison to existing literature. Prior studies often use more specific criteria (e.g., stereopsis ≤100 arcsec) for sensory success. The authors should clearly define the threshold used, justify it in the context of the literature, and acknowledge any limitations if a less stringent criterion was applied.
Response: Thank you for your valuable suggestion. We apologize for the incorrect mention of the success of sensory outcomes. The sensory status only referred to the detection of stereopsis, which was identified by the near stereoacuity test, but it was not a successful sensory outcome. Thank you for your insightful comment. The sentence was rewritten for greater clarity and a more academic tone, as suggested on pages 5-6, lines 122-124. Furthermore, the success of sensory status in the manuscript was clarified through rewriting, and the term “stereopsis detection” was replaced accordingly on page 13, line 271.

Definition of “Absent” Stereopsis in Table 2:
Table 2 refers to stereopsis as "present" or "absent," but the manuscript does not define what constitutes “absent” stereopsis. The authors should specify whether this refers to a complete lack of measurable stereopsis on the Randot test, and clarify the cutoff or conditions under which stereopsis was considered absent.

Response: Thank you for your valuable suggestion. The sensory status was only the detection of any stereopsis, not a confirmation of successful sensory function. Thank you for your excellent comment. The sentence was revised for greater clarity and a more academic tone, as suggested, in the method section of the revised manuscript, pages 5-6, lines 122-124.

Reviewer #2: Dear Authors,

Thank you for the opportunity to review this interesting and clinically relevant manuscript. The study investigates surgical outcomes in intermittent exotropia (IXT) over a 9-year period, which is important for both regional and global understanding of treatment success and predictive factors. The manuscript is well-structured, and the research question is clearly defined. However, several methodological and reporting aspects need clarification and improvement to enhance the validity and reproducibility of your findings.

A. Major Concerns
1. Definition of Postoperative Consecutive Esotropia (CE): Since early CE was identified as a significant predictor of surgical success, please clarify the clinical cutoff used to define CE (e.g., >5 PD or >10 PD). A precise definition is essential for reproducibility and for clinicians to apply these findings in practice.

Response: Thank you for your valuable suggestion. The exact definition of surgical success has been written. The cut-off value for early consecutive esotropia > 5 prism diopters was noted in the method section of the revised manuscript on page 5, lines 121-122.

2. Numerical Inconsistency in Table 3: In Table 3 (Day 1), the total number of patients across alignment categories does not equal 150. Please verify whether this is due to missing follow-up data or a reporting error, and provide an explanation or corrected table as appropriate.

Response: Thank you for your valuable suggestion. We apologize for an error in data presentation. Thank you for your careful evaluation. The reporting error was corrected to reflect exactly 150 total cases at each point. Table 3 was rechecked, and the correct numbers were inserted into each cell.

3. Sample Size vs Study Duration:Over a 9-year period, a sample size of 150 appears relatively small for a tertiary center. Please clarify whether this was due to strict eligibility criteria, loss to follow-up, or exclusion based on comorbidities or incomplete records. Discuss any resulting implications for generalizability and statistical power in your limitations.

Response: Thank you for your valuable suggestion. The small sample size was due to the study's strict inclusion criteria. Most patient losses to follow-up aligned with the inclusion criteria, resulting in a small number of participants. The discussion was mentioned in the limitations part of the manuscript on page 16, lines 356-357.

4. Handling of Missing Stereopsis Data: Only 131 of 150 participants had preoperative stereopsis data. Please clarify how missing data were handled in the logistic regression analysis (e.g., complete-case analysis, imputation, or exclusion).

Response: Thank you for this valuable comment. We acknowledge that preoperative stereopsis data were missing for 19 participants. For the logistic regression analysis, we performed a complete-case analysis, excluding cases with missing stereopsis data from the model. This approach was chosen to avoid potential bias from imputation and to ensure the robustness of the regression estimates. The final analysis included only participants with complete stereopsis data. The handling of missing stereopsis data excluded from logistic regression was added in the methods section of the revised manuscript on page 7, lines 147-151.

5. Statistical Model Reporting: The multivariate logistic regression is well-conceived, but the analysis lacks model validation metrics. Consider adding or commenting on:
a. Goodness-of-fit test (e.g., Hosmer-Lemeshow).
b. Discrimination (e.g., AUC/ROC curve).
3. Multicollinearity checks.
These would strengthen confidence in the identified predictors.

Response: Thank you for this insightful comment. In the revised manuscript, we have added model validation metrics to strengthen the robustness of our multivariable logistic regression findings. Specifically, we performed the Hosmer–Lemeshow goodness-of-fit test, demonstrating adequate model calibration (p > 0.05). We also assessed model discrimination using the area under the receiver operating characteristic (ROC) curve, which showed acceptable discriminatory ability. In addition, multicollinearity was evaluated using variance inflation factors (VIFs), and no concern for multicollinearity was identified (all VIFs < 2). These details have been incorporated into the Statistical Analysis section (page 8, lines 152–155) and the Results section (page 12, lines 249–251) of the revised manuscript.

6. Definition of Success in Tables: Please reiterate your criteria for motor and sensory success in the footnotes of Tables 2–4. This will improve clarity and consistency for the reader.

Response: Thank you for your valuable suggestion. The definition of motor success and detection of stereopsis status was noted in the footnote of Table 2-4 to improve clarity for readers.

7. Surgeon Variability: Since three surgeons performed procedures over the study period, please clarify whether inter-surgeon variability was assessed or considered as a confounding factor.

Response: Thank you for this valuable suggestion. We considered inter-surgeon variability as a potential confounding factor. While the study was not powered to perform a detailed stratified analysis by individual surgeon, we acknowledge this variability and have discussed it as a limitation in the revised manuscript (page 16, lines 351-353), noting that differences in surgical technique or experience could influence the generalizability of our findings.

8. references : most of the references are before 2020, I would request authors to look into new research articles and comparison with recent literature.

Response: Thank you for this helpful suggestion. We have updated the reference list in the revised manuscript to include newer studies and ensure comparison with current literature. We appreciate your thoughtful recommendation to enhance the manuscript’s relevance and context. The additional references were reference numbers 7, 8, and 10.

B. Minor Suggestions
1. Please ensure Figures 1 and 2 are included and fully labeled in the revised manuscript. Their absence currently limits interpretation of key findings.

Response: Thank you for your valuable suggestion. Figures 1 and 2 were included and properly labeled in the revised manuscript as suggested. Thank you for your thorough review.

2. You may consider including a visual summary (e.g., boxplot or bar graph) of surgical outcomes stratified by presence/absence of stereopsis and CE.

Response: Thank you for this valuable suggestion. We have added a visual summary illustrating the surgical outcomes stratified by the presence or absence of stereopsis and consecutive esotropia to enhance the clarity of our findings. This figure has been included as Figure 4 in the revised manuscript.

3. If feasible in future studies, include distant stereopsis testing, as IXT control is often more relevant at distance fixation.

Response: Thank you for your valuable suggestion. The limitation of the study was noted and the future studies are advised to incorporate the distance stereopsis in the study per comment on page 16, lines 361-363.

Finally in conclusion,this study provides valuable insight into IXT management and postoperative outcomes in a population that is underrepresented in the literature. I would like encourage revision to clarify definitions, correct inconsistencies, and improve the statistical reporting. With these revisions, the manuscript will offer meaningful contributions to the field of pediatric and strabismus surgery.

Best regards

Response: Thank you for your valuable suggestion and the time you have dedicated to reviewing our manuscript. We have thoroughly revised the manuscript to address the reviewers’ comments and enhance its clarity and scientific quality. We hope that these revisions, together with our detailed responses, will satisfactorily resolve the concerns raised and render the manuscript suitable for publication in the journal.

Reviewer #3: As the statistical reviewer I will focus on methods and reporting.

Major
1) the sample is small and power will be limited, especially in a multivariable regression framework. findings need to be discussed carefully and not as definitive.

Response: Thank you for your valuable suggestion. The small sample size is a limitation of the study and may reduce the study's power. I discuss this thoroughly in the points on the limitation part of the manuscript on page 16, lines 356-357. The small sample size may limit power and affect the likelihood of obtaining definitive positive results in our study.

2) the methods section needs to be written with more clarity about the comparisons made, e.g. in what variables and for what purposed.

Response: Thank you for this helpful comment. We have revised the Methods section to provide greater clarity regarding the comparisons performed in this study. Specifically, we now explicitly describe which variables were compared (e.g., demographic, clinical, and surgical factors), the time points of comparison (preoperative vs. postoperative outcomes), and the purpose of these comparisons (e.g., to evaluate changes in alignment, assess stereopsis improvement, and identify predictors of surgical success). These revisions are reflected in the revised manuscript (Methods section, page 7, lines 131–137).

3) the STROBE statement has been submitted but I cannot find information as to how missing data were handled. Considering levels of missingness, why weren't multiple imputation approaches considered?

Response: Thank you for this useful comment. We acknowledge that preoperative stereopsis data were missing for 17 participants. For the logistic regression analysis, we used a complete-case approach, excluding cases with missing stereopsis data from the model. We chose this method over multiple imputation because the proportion of missing data was relatively low (11.3%) and the missingness was not expected to cause systematic bias. This approach also helped ensure the robustness and transparency of the regression estimates. Details on how missing stereopsis data were handled have been added to the revised Methods section (page 7, lines 147–151).

4) Near stereopsis outcome has 3 categories and it was not clear if and how analysed.

Response: Thank you for your valuable suggestion. I have removed the near stereopsis category in the revised manuscript as per your comment. My original intention was to analyze whether the level of stereopsis influences the success of surgery; however, the results were negative. No association was found between preoperative stereopsis status and surgical success in the subgroup analysis.

Minor
1) multivariable not multivariate (multiple outcomes) regression.

Response: Thank you for your valuable suggestion. The incorrect word was revised throughout the manuscript, on pages 7, 12, 12, lines 143, 235, and 240.

2) how were variables selected for inclusion in the multivariable logistic regression

Response: Thank you for this insightful comment. Variables included in the multivariable logistic regression model were selected a priori based on both their statistical significance in univariable analysis and their established clinical relevance as potential predictors of surgical success (e.g., visual acuity, preexisting stereopsis, presence of consecutive esotropia at one week, and preoperative strabismic angle). This rationale and selection process are now explicitly stated in the Methods section of the revised manuscript (page 7, lines 145–147).

6. PLOS authors have the option to publish the peer review history of their article ([what does this mean?](https://track.editorialmanager.com/CL0/https:%2F%2Fjournals.plos.org%2Fplosone%2Fs%2Feditorial-and-peer-review-process%23loc-peer-review-history/1/010f01986132418c-5438627a-5149-4859-bc90-07b8441783a9-000000/E_QWN4jCqHaXSFxeFLlym-s57V0by-VNWSGVxMv8R1o=222)). If published, this will include your full peer review and any attached files.


If you choose “no”, your identity will remain anonymous but your review may still be made public.


**Do you want your identity to be public for this peer review?** For information about this choice, including consent withdrawal, please see our [Privacy Policy](https://track.editorialmanager.com/CL0/https:%2F%2Fwww.plos.org%2Fprivacy-policy/1/010f01986132418c-5438627a-5149-4859-bc90-07b8441783a9-000000/bFLKxqbQH5xo1tCyPfmcGoPA3TRMlSRk5YaOraRe1Pg=222).

Reviewer #1: **Yes:** HYUN JIN SHIN

Reviewer #2: No

Reviewer #3: No

[NOTE: If reviewer comments were submitted as an attachment file, they will be attached to this email and accessible via the submission site. Please log into your account, locate the manuscript record, and check for the action link "View Attachments". If this link does not appear, there are no attachment files.]

While revising your submission, please upload your figure files to the Preflight Analysis and Conversion Engine (PACE) digital diagnostic tool, [https://pacev2.apexcovantage.com/](https://track.editorialmanager.com/CL0/https:%2F%2Fpacev2.apexcovantage.com%2F/1/010f01986132418c-5438627a-5149-4859-bc90-07b8441783a9-000000/OT8eG0e1E5uoSoOjOfBDvPhuweZfgM7dDFaoqvjVl-Y=222). PACE helps ensure that figures meet PLOS requirements. To use PACE, you must first register as a user. Registration is free. Then, login and navigate to the UPLOAD tab, where you will find detailed instructions on how to use the tool. If you encounter any issues or have any questions when using PACE, please email PLOS at [figures@plos.org](mailto:figures@plos.org). Please note that Supporting Information files do not need this step.
