# Supplementary material for: Early postoperative alignment and preoperative stereopsis as determinants of success in intermittent exotropia surgery
Source: PLoS One. 2025 Oct 10;20(10):e0329609. doi: 10.1371/journal.pone.0329609 (PMC12513599; doi:10.1371/journal.pone.0329609)
Supplement: Attachment — Submitted filename: Response_to_Reviewers_auresp_2.docx [file pone.0329609.s005.docx]

**Response letter (Revision R2)**

PLOS One

PONE-D-25-34023R1

Early Postoperative Alignment and Preoperative Stereopsis as Determinants of Success in Intermittent Exotropia Surgery

PLOS ONE

Dear Dr. Srimanan,

Thank you for submitting your manuscript to PLOS ONE. After careful consideration, we feel that it has merit but does not fully meet PLOS ONE’s publication criteria as it currently stands. Therefore, we invite you to submit a revised version of the manuscript that addresses the points raised during the review process.

Please submit your revised manuscript by Oct 31 2025 11:59PM. If you will need more time than this to complete your revisions, please reply to this message or contact the journal office at [plosone@plos.org](mailto:plosone@plos.org). When you're ready to submit your revision, log on to <https://www.editorialmanager.com/pone/> and select the 'Submissions Needing Revision' folder to locate your manuscript file.

Please include the following items when submitting your revised manuscript:

- A rebuttal letter that responds to each point raised by the academic editor and reviewer(s). You should upload this letter as a separate file labeled 'Response to Reviewers'.
- A marked-up copy of your manuscript that highlights changes made to the original version. You should upload this as a separate file labeled 'Revised Manuscript with Track Changes'.
- An unmarked version of your revised paper without tracked changes. You should upload this as a separate file labeled 'Manuscript'.

If you would like to make changes to your financial disclosure, please include your updated statement in your cover letter. Guidelines for resubmitting your figure files are available below the reviewer comments at the end of this letter.

If applicable, we recommend that you deposit your laboratory protocols in [protocols.io](http://protocols.io/) to enhance the reproducibility of your results. Protocols.io assigns your protocol its own identifier (DOI) so that it can be cited independently in the future. For instructions see: [https://journals.plos.org/plosone/s/submission-guidelines#loc-laboratory-protocols](https://track.editorialmanager.com/CL0/https:%2F%2Fjournals.plos.org%2Fplosone%2Fs%2Fsubmission-guidelines%23loc-laboratory-protocols/1/010f019950b42eeb-713beab1-e8b6-45e0-894e-f5d5c85368cc-000000/JQj9zlzuGCgNDQ2xRH_Jadl4MLrtAHjGaV87p_g6FB4=228). Additionally, PLOS ONE offers an option for publishing peer-reviewed Lab Protocol articles, which describe protocols hosted on [protocols.io](http://protocols.io/). Read more information on sharing protocols at [https://plos.org/protocols?utm_medium=editorial-email&utm_source=authorletters&utm_campaign=protocols](https://track.editorialmanager.com/CL0/https:%2F%2Fplos.org%2Fprotocols%3Futm_medium=editorial-email%26utm_source=authorletters%26utm_campaign=protocols/1/010f019950b42eeb-713beab1-e8b6-45e0-894e-f5d5c85368cc-000000/p3MDVnANvBZGmzZdmMgaQzM0HWioVtsxBAuVk07rau8=228).

We look forward to receiving your revised manuscript.

Kind regards,

PremNandhini Satgunam
Academic Editor
PLOS ONE

**Journal Requirements:**

If the reviewer comments include a recommendation to cite specific previously published works, please review and evaluate these publications to determine whether they are relevant and should be cited. There is no requirement to cite these works unless the editor has indicated otherwise. 

**Additional Editor Comments:**

Dear Authors,

All the reviewers have received your revision positively. However, one reviewer is pointing to some methodological enhancements, which I believe will strengthen your paper. Kindly address those concerns and submit a revision.

Response: Dear Editor,

Thank you very much for your continued consideration of our manuscript and for sharing the additional feedback from the reviewers.

We sincerely appreciate the reviewer’s insightful suggestions regarding methodological enhancements. We have carefully addressed the remaining concerns and revised the Methods and related sections of the manuscript accordingly to strengthen the rigor and transparency of our study.

We believe these revisions have further improved the clarity and scientific contribution of our work, and we are grateful for the opportunity to refine the manuscript to better meet the standards of your journal.

Thank you once again for your guidance and for the opportunity to resubmit our revised manuscript.

Sincerely,
Worapot Srimanan, MD
(On behalf of all co-authors)

[Note: HTML markup is below. Please do not edit.]

Reviewers' comments:

Reviewer's Responses to Questions

**Comments to the Author**

1. If the authors have adequately addressed your comments raised in a previous round of review and you feel that this manuscript is now acceptable for publication, you may indicate that here to bypass the “Comments to the Author” section, enter your conflict of interest statement in the “Confidential to Editor” section, and submit your "Accept" recommendation.

Reviewer #1: All comments have been addressed

Reviewer #2: All comments have been addressed

Reviewer #3: All comments have been addressed

2. Is the manuscript technically sound, and do the data support the conclusions?

The manuscript must describe a technically sound piece of scientific research with data that supports the conclusions. Experiments must have been conducted rigorously, with appropriate controls, replication, and sample sizes. The conclusions must be drawn appropriately based on the data presented.

Reviewer #1: Yes

Reviewer #2: Partly

Reviewer #3: Yes

3. Has the statistical analysis been performed appropriately and rigorously?

Reviewer #1: Yes

Reviewer #2: Yes

Reviewer #3: Yes

4. Have the authors made all data underlying the findings in their manuscript fully available?

The [PLOS Data policy](http://track.editorialmanager.com/CL0/http:%2F%2Fwww.plosone.org%2Fstatic%2Fpolicies.action%23sharing/1/010f019950b42eeb-713beab1-e8b6-45e0-894e-f5d5c85368cc-000000/dEfXUYc09y5MgSPc-Nd8EkW9EpBWLjhRGFe2Kdw0Dnk=228) requires authors to make all data underlying the findings described in their manuscript fully available without restriction, with rare exception (please refer to the Data Availability Statement in the manuscript PDF file). The data should be provided as part of the manuscript or its supporting information, or deposited to a public repository. For example, in addition to summary statistics, the data points behind means, medians and variance measures should be available. If there are restrictions on publicly sharing data—e.g. participant privacy or use of data from a third party—those must be specified.

Reviewer #1: Yes

Reviewer #2: Yes

Reviewer #3: Yes

5. Is the manuscript presented in an intelligible fashion and written in standard English?

PLOS ONE does not copyedit accepted manuscripts, so the language in submitted articles must be clear, correct, and unambiguous. Any typographical or grammatical errors should be corrected at revision, so please note any specific errors here.

Reviewer #1: Yes

Reviewer #2: Yes

Reviewer #3: Yes

6. Review Comments to the Author

Please use the space provided to explain your answers to the questions above. You may also include additional comments for the author, including concerns about dual publication, research ethics, or publication ethics. (Please upload your review as an attachment if it exceeds 20,000 characters)

**Reviewer #1:** I appreciate for your detailed responses. Your answer has resolved my questions. Thank you for revising the paper so well.

Response: We sincerely thank you for your insightful and constructive feedback. Your comments were invaluable in strengthening our manuscript and enhancing its scientific rigor. We are truly grateful for the time and careful effort you dedicated to reviewing our work.

**Reviewer #2:** Dear Authors,
Thank you for submitting your first revision. The topic is clinically meaningful, and your focus on early postoperative alignment and preoperative stereopsis is valuable for surgical decision-making in intermittent exotropia (IXT). I appreciate the clarified abstract, clear success definition, and the expanded description of stereopsis testing in the comments.

To plan towards acceptance, I’ve tried some more suggestions followed by optional refinements. Several items are straightforward clarifications and formatting, while a few require adding core methodological and statistical details so readers can appraise your findings.

1. Methods and reporting (STROBE): Please ensure the Methods and Results adhere to the STROBE checklist for observational studies, with complete reporting of setting, participants, variables and definitions, data sources/measurement, bias, study size, statistical methods (including handling of confounders, missing data, and sensitivity analyses), participant flow, and outcome reporting.

Response: Thank you very much for your valuable and constructive comment. We have reviewed and revised our manuscript per the STROBE (Strengthening the Reporting of Observational Studies in Epidemiology) checklist to enhance methodological transparency and rigor.

We have made the following improvements:

- Clearly described the study setting and participant inclusion criteria, with definitions of all relevant variables and outcomes (Methods section);
- Specified data sources and measurement techniques, particularly for stereopsis and postoperative alignment assessment;
- Addressed potential sources of bias inherent to the retrospective design and described strategies to minimize them;
- Provided details on sample size, missing data handling, and the statistical methods used, including multivariable logistic regression to control for potential confounders;
- Documented participant flow from initial chart review to final analysis and presented outcome distributions across all timepoints (Results section).

Additionally, we have included the completed STROBE checklist with this resubmission to demonstrate explicit adherence to all recommended reporting elements.

We sincerely appreciate your guidance, which has helped us improve the clarity, completeness, and transparency of our reporting.

2. Control score and neurologic status:
Include the IXT control score in the Methods (scale used, distance vs. near, measurement protocol) and report how neurologic status was assessed and handled (inclusion/exclusion criteria and any subgroup analyses).

Response: Thank you for your thoughtful and constructive comment. The control of intermittent exotropia was assessed during clinical follow-up visits using a standard office-based scale that evaluated both near and distance control. This evaluation contributed to the surgical decision-making process. However, as this study was retrospective in nature, documentation of the control score was found to be inconsistent and incomplete in patient records. As a result, this variable was not included in the outcome analysis or multivariable regression model. We have clarified this point in the revised Methods section and acknowledged it as a limitation of the study, on page 16, lines 365-367.

With regard to neurologic status, we confirm that a formal neurologic assessment was not performed or recorded in this study. No specific neurologic inclusion or exclusion criteria were applied, and subgroup analysis based on neurologic status could not be conducted. This has now been explicitly noted in the Discussion section as a limitation, on page 16, lines 367-369.

3. Multivariate analysis: Add multivariable analyses that adjust for age (2-66 yrs) and control (and other clinically relevant covariates), and report adjusted estimates with 95% confidence intervals. This will strengthen the evidence for the proposed predictors of surgical success.
Response: We sincerely appreciate this valuable suggestion. Per your recommendation, we have revised the multivariable logistic regression analysis to include age as an additional covariate. The updated results are presented in Table 4 of the revised manuscript, and relevant revisions have been made throughout the Results, Methods, and Discussion sections. (Page 7, line 146)

While we acknowledge the importance of the control score as a potential predictor, this variable was excluded from our regression model due to inconsistent documentation in the medical records, as outlined above. This has been noted clearly as a limitation of the study in the revised manuscript, on page 16, lines 365-367.

4. Table 3- (postoperative surgical alignment), the last three rows require additional explanation. The deviation range is noted as 0–25 PD, yet the cohort includes patients with consecutive esotropia. Please justify the displayed ranges, clarify how esodeviations were coded (e.g., negative values), and revise axis labels/legends accordingly to avoid ambiguity.

Response: Thank you for this insightful comment. We appreciate the opportunity to clarify this important point.

In our analysis, we reported the magnitude of strabismic deviation in positive prism diopters (PD) regardless of direction (i.e., both exodeviation and esodeviation are represented as positive values). For clarity and consistency, patients with consecutive esotropia were included in a separate “consecutive esotropia” category, and their angle of deviation was recorded as a positive value corresponding to the measured esodeviation.

To avoid confusion, we have now added a specific note to the Table 3 legend to explain this coding approach, emphasizing that angle values reflect absolute magnitudes in PD, not directional values (positive for exo or negative for eso). We have also clarified the range of deviations and removed directional ambiguity from the axis labels where applicable.

5. Standardize terms for success, failure/recurrence, and overcorrection; define how diplopia was ascertained (symptom report vs. clinical test).

Response: Thank you for your insightful comment. In response, we have defined key terms relevant to our outcome measures—such as surgical success, orthotropia, consecutive esotropia, and overcorrection—in the Methods section of the revised manuscript. However, we did not report failure or recurrence as predefined outcome measures in this study, and therefore, these terms were intentionally omitted to maintain consistency and clarity in the manuscript.

Diplopia was assessed based on subjective symptom reports provided by patients during clinical follow-up visits rather than through formal clinical testing. This has now been clarified in both the Methods and Disease Definitions sections of the revised manuscript. (on page 5, lines 122-124)

Optional refinements

1. Specify the primary outcome timepoint (e.g., 12 months or last follow-up) and provide follow-up distribution (median, IQR).

Response: Thank you for your valuable and constructive comment. We have clarified that the primary outcome time point was assessed at 12 months postoperatively; this has been explicitly stated in the revised Methods section of the manuscript.

Additionally, the follow-up duration has been described in accordance with your suggestion. Specifically, the median follow-up was 14.3 months, with an interquartile range (IQR) of 12.6–15.8 months, as now included in the Results section, on page 8, lines 171-172.

We appreciate your recommendation, which has helped enhance the clarity and completeness of our manuscript.

2. If feasible, include time-to-event analyses for recurrence or failure to characterize durability.

Response: Thank you for your insightful and constructive suggestion. We fully agree that time-to-event analyses (e.g., Kaplan–Meier or Cox regression) could provide valuable information regarding the durability of surgical outcomes. However, due to the retrospective nature of our dataset and the limited availability of precise time points for recurrence or failure across all participants, we could not perform a robust time-to-event analysis. We acknowledge this as a limitation of our study and have clarified this point in the revised Discussion section, on page 16, lines 369-371.

I appreciate the progress made in this revision. Addressing the items above will materially improve clarity, transparency, and the interpretability of your findings.

Sincerely

**Reviewer #3:** I am satisfied with the authors' responses and the resulting changes to the paper. I have nothing further to add.

Response: We would like to express our sincere gratitude to you for your thoughtful review and the considerable effort you made to improve our paper. Your expertise was instrumental in enhancing the quality of our work.

7. PLOS authors have the option to publish the peer review history of their article ([what does this mean?](https://track.editorialmanager.com/CL0/https:%2F%2Fjournals.plos.org%2Fplosone%2Fs%2Feditorial-and-peer-review-process%23loc-peer-review-history/1/010f019950b42eeb-713beab1-e8b6-45e0-894e-f5d5c85368cc-000000/akOl2Cn8VOqg2y1SCAVJdeUcr_wkOazK1MQWz3qJcKI=228)). If published, this will include your full peer review and any attached files.


If you choose “no”, your identity will remain anonymous but your review may still be made public.


**Do you want your identity to be public for this peer review?** For information about this choice, including consent withdrawal, please see our [Privacy Policy](https://track.editorialmanager.com/CL0/https:%2F%2Fwww.plos.org%2Fprivacy-policy/1/010f019950b42eeb-713beab1-e8b6-45e0-894e-f5d5c85368cc-000000/ZQ2laV_wz_7Oq3xv3eugBLlQQVuse1nY-yZW1mvDr3I=228).

Reviewer #1: No

Reviewer #2: **Yes:** Sampada Kulkarni

Reviewer #3: No

[NOTE: If reviewer comments were submitted as an attachment file, they will be attached to this email and accessible via the submission site. Please log into your account, locate the manuscript record, and check for the action link "View Attachments". If this link does not appear, there are no attachment files.]

While revising your submission, please upload your figure files to the Preflight Analysis and Conversion Engine (PACE) digital diagnostic tool, [https://pacev2.apexcovantage.com/](https://track.editorialmanager.com/CL0/https:%2F%2Fpacev2.apexcovantage.com%2F/1/010f019950b42eeb-713beab1-e8b6-45e0-894e-f5d5c85368cc-000000/wp73CRuuUs2tLqLDUHp4Icq-43p8hAQ9MkeZJ3zJrf0=228). PACE helps ensure that figures meet PLOS requirements. To use PACE, you must first register as a user. Registration is free. Then, login and navigate to the UPLOAD tab, where you will find detailed instructions on how to use the tool. If you encounter any issues or have any questions when using PACE, please email PLOS at [figures@plos.org](mailto:figures@plos.org). Please note that Supporting Information files do not need this step.
